# Oxidative Stress on the Ground and in the Microgravity Environment: Pathophysiological Effects and Treatment

**DOI:** 10.3390/antiox14020231

**Published:** 2025-02-18

**Authors:** Xinyuan Zhang, Huaiying Zhu, Jinhua Zhang

**Affiliations:** 1 State Key Laboratory of Targeting Oncology, National Center for International Research of Bio-targeting Theranostics, Guangxi Key Laboratory of Bio-targeting Theranostics, Collaborative Innovation Center for Targeting Tumor Diagnosis and Therapy, Guangxi Talent Highland of Major New Drugs Innovation and Development, Guangxi Medical University, Nanning 530021, China; 202221563@sr.gxmu.edu.cn (X.Z.); zhuhy-05@sr.gxmu.edu.cn (H.Z.); 2 College of Life Science and Bioengineering, Beijing Jiaotong University, Beijing 100044, China

**Keywords:** oxidative stress, microgravity, reactive oxygen species, antioxidants, therapy

## Abstract

With the continued exploration of the universe, there is an increasingly urgent need to address the health challenges arising from spaceflight. In space, astronauts are exposed to radiation, confinement and isolation, circadian rhythm dysregulation, and microgravity conditions that are different from those on Earth. These risk factors jeopardize astronauts’ health, thus affecting the quality of space missions. Among these factors, gravitational changes influence the balance between oxidation and antioxidants, stimulating the production of reactive oxygen species (ROS), finally leading to oxidative stress (OS). OS leads to oxidative damage of biomolecules such as lipids, proteins, and DNA, which causes the development of various diseases. The occurrence of OS is increased in microgravity and affects multiple systems, including the musculoskeletal, cardiovascular, nervous, and immune systems. In this review, we discuss the mechanisms of OS, the physiological effects on different systems caused by OS in microgravity environment, and potential treatments for OS. Finally, treatment strategies for oxidative stress in microgravity are summarized, providing some promising approaches for protecting the health of astronauts in future space exploration.

## 1. Introduction

Since the first human spaceflight, the exploration of the universe has never stopped, and the number of astronauts, the frequency of flights, and the duration of their missions are gradually increasing. Resolving health issues during spaceflights is becoming an urgent priority. In space, astronauts are exposed to space radiation, microgravity and altered gravity, confinement and isolation, hostile and closed environments, medical limitations, and other spaceflight risk factors. These factors compromise the health of astronauts, thus affecting the quality of space missions [1]. Among these factors, microgravity plays an important role that cannot be ignored. It causes structural and functional changes in the musculoskeletal, nervous, cardiovascular, digestive, and immune systems, among others, exposing astronauts to a range of health risks. In microgravity, space adaptation syndrome (SAS) is triggered in the early stages [2]. This syndrome can trigger symptoms such as nausea, disorientation, and generalized malaise, which typically last up to 72 h [3]. Due to the lack of downward attraction from gravity, body fluids are transferred to the upper body and head, increasing intracranial pressure (ICP) or optic nerve compression in the eyes [4]. This leads to retinal endothelium dysfunction, in turn causing several ophthalmic symptoms, including choroidal folds, optic disc edema, and cotton-wool spots [5]. This phenomenon has been summarized by Sara et al. as spaceflight-associated neuro-ocular syndrome (SANS) [6]. The transfer of body fluids to the head causes changes in the brain white matter microstructure [7], developing a hydrocephalus-like structure, which damages brain tissue and impairs brain function [8]. The microgravity environment also impairs bones and skeletal muscles, contributing to conditions such as osteoporosis and skeletal muscle atrophy [9,10]. In microgravity, the gut microbiota is dysregulated, the intestinal mucosal barrier is compromised, and mucosal permeability is increased [11]. As a result, the stability of the intestinal microenvironment and gastrointestinal dynamics is compromised, triggering local or systemic reactions [12]. The immune system function is also disrupted under the influence of microgravity, leading to phenomena such as an inhibition of T-cell antigen reactivity and a rise in white blood cell counts, neutrophils, and monocytes [13,14]. Currently, there are limited studies related to the molecular mechanisms involved in these symptoms caused by microgravity.

Exogenous risk factors such as radiation, tobacco, and drugs disrupt the balance between oxidation and antioxidants, stimulating the production of reactive oxygen species (ROS) and finally leading to oxidative stress (OS) [15]. Moreover, OS occurs in a variety of organs and cells, causing tissue damage and dysfunction in microgravity. Microgravity promotes the expression of oxidative enzymes, such as nicotinamide adenine dinucleotide phosphate (NADPH) oxidases (NOXs) [16]. NOXs expression is increased in the brain, skeletal muscles, blood vessels, and lungs in microgravity [17,18,19,20]. Microgravity also reduces the expression of antioxidant proteins, such as nuclear transcription factor red lineage 2-related factor 2 (Nrf2), which increases oxidative products and leads to OS [21]. Nrf2, a transcription factor, is essential to the OS response, as it binds to antioxidant response elements (AREs) and regulates antioxidant proteins [22]. Nrf2 abundance was reduced significantly in the flounder muscle of rats by using hindlimb unloading (HLU) to simulate microgravity, leading to muscle atrophy [19]. Although microgravity-induced OS causes difficulties for astronauts in space operations, the molecular mechanisms and treatments of OS in microgravity have been less well investigated.

This review summarizes the physiological changes in systems caused by OS in the microgravity environment, describes the treatments of OS both on the ground and in microgravity, and elucidates the health effects of OS in the microgravity environment. It provides some new ideas for future research on OS and for treating organ damage caused by OS in space in the future. This review provides a preliminary perspective on OS in space for the field, thereby laying the foundation for more focused future research. We collected relevant references through an extensive literature search to improve the comprehensiveness and authority of this review. Future research needs to explore more specific aspects to address the gaps identified in the current study.

## 2. Oxidative Stress

### 2.1. Mechanisms of Oxidative Stress

OS was first described in a study in 1985 [23]. OS occurs when the production and accumulation of ROS exceed the capacity of the antioxidant defense, resulting in a disruption of the metabolic homeostasis of the organism [24]. OS is characterized by a disruption of the oxidant–antioxidant balance. ROS, including superoxide anion (O_2_^•−^), hydrogen peroxide (H_2_O_2_), and hydroxyl radicals (•OH), lead to oxidative damage to lipids, proteins, and DNA in cells, triggering tissue damage [25]. To illustrate this, DNA damage was caused by the ROS-induced oxidation of guanine to pre-mutagenic 8-oxo-7,8-dihydroguanine (8-oxoG), which resulted in double-strand breaks during replication; proteins were misfolded due to exposure to ROS, causing changes in intramolecular interactions [26].

The production of ROS involves various factors. Mitochondria produce ROS during oxidative phosphorylation (OXPHOS) [27]. Similarly, enzymes such as NADPH oxidases (NOXs) also contribute to ROS production [28]. Additionally, ROS levels increased when the body underwent pathological changes, such as inflammatory reactions and ischemia/reperfusion injury [29,30]. Exogenous risk factors, including environmental pollutants, radiation, drugs, and tobacco, also directly or indirectly increased ROS production [15].

As well as increased ROS production, ROS accumulation due to an unregulated antioxidant system is also an important cause of OS. Antioxidant enzymes, metal ion chelators, small-molecule antioxidant compounds, and enzymes that play a role in repairing oxidative damage are all important components of the antioxidant system [25]. Antioxidant enzymes include superoxide dismutase (SOD), catalase (CAT), peroxidase (POD), and ascorbate peroxidase (APX) [31]. Typically, antioxidant enzymes are activated through Nrf2 and PGC-1α signaling pathways [22]. Metal ion chelators bind to transition metal ions, thereby reducing the generation of free radicals catalyzed by these ions. For instance, ferritin and transferrin store and transport iron ions, preventing these ions from catalyzing oxidative reactions [32]. Furthermore, small-molecule antioxidant compounds, including ascorbic acid (vitamin C), alpha-tocopherol (vitamin E), glutathione (GSH), carotenoids, and flavonoid analogs, directly reacted with ROS to scavenge free radicals [33]. DNA damage repair-related enzymes, such as formamidopyrimidine DNA glycosylase (FPG) and 8-oxoguanine DNA glycosylase (OGG1), repair damage caused by OS [34]. In conclusion, the dysregulation of the antioxidant system results in the inability to rapidly scavenge ROS and repair damage from OS in a timely manner, leading to the development of diseases in organisms.

### 2.2. Effects of OS on the Ground

OS is a key factor in the development of various diseases on the ground. In some diseases, OS is a major factor in pathologies such as radiation-induced lung injury, paraquat poisoning, and atherosclerosis [25]. For instance, in a radiation environment, water molecules produced ^•^OH, which led to an increase in oxidative intermediates, inflammation, and, ultimately, lung injury [35]. In the majority of cases, OS is an indirect factor in disease progression. The excessive production of ROS inhibits cell proliferation; accelerates cellular senescence; and induces senescence-related diseases, including retinal diseases, neurodegenerative diseases, osteoporosis, cardiovascular diseases, and cancers [36,37,38,39,40]. Moreover, ROS accumulation in the bone microenvironment was found to play a role in osteoblast and osteoclast apoptosis [41]. Research has also shown that ROS was increased in the rheumatoid arthritis-associated synovial microenvironment under hypoxia. A high-ROS-level environment caused fibroblast proliferation and the secretion of inflammatory factors by macrophage-like synoviocytes and increased angiogenesis, leading to cellular and tissue damage, driving disease progression [42]. In addition, a close interaction was found between OS and the immune response. ROS activated the immune response and promoted the release of cytokines, while immune cells produced ROS when clearing pathogens, which killed part of them [43]. However, excessive ROS inhibited immune activity and suppressed signal transduction between dendritic cells (DCs) and T cells, affecting the normal function of the immune system [44,45]. Matsushita et al. found that the lipid peroxidation of T cells induced iron death and prevented the immune response to infection [46]. In brief, on the ground, OS plays a crucial role in diseases by directly or indirectly affecting various organs and systems on the ground.

## 3. Effects of OS on the Organism in Microgravity

This section summarizes in vitro and in vivo experimental data from studies about microgravity to describe the effects of microgravity-induced OS on biological samples. Experiments in real microgravity environments are typically carried out during spaceflight, such as on rockets, on the ISS, and in parabolic flight [47]. However, experiments in space are costly, and the duration is limited by the work schedules. Therefore, experiments in simulated microgravity on the ground have become an important method [48]. Both in vivo and in vitro models have been available to simulate microgravity for experimental protocols. Among the in vivo models are HLU, long-duration bed rest, and dry immersion [49,50,51]. Additionally, in vitro models such as rotating wall vessels, the random positioning machine, and the 3D clinostat are also available for use [52]. These methods have various advantages and limitations, and each method has its unique value, which will not be elaborated upon here. However, different methods introduce variability in the experimental results. Therefore, we have listed the models summarizing the experimental data in tables.

### 3.1. Effects of OS on the Musculoskeletal System in Microgravity

Microgravity has a significant impact on the musculoskeletal system. On the ground, bones are stimulated by mechanical loads, new bone is generated to replace old or damaged bones, and bones are constantly remodeled, creating a balance [53]. This balance is broken by microgravity, which sharply reduces the mechanical loads on bones and muscles. New bone production slows down due to lack of stimulation, leading to bone loss [10,54]. Microgravity regulates the distribution and content of minerals in the body in many ways, such as altering the absorption and excretion processes of calcium and magnesium and changing the electrolyte balance [55]. The effect of changing the electrolyte balance on skeletal muscles is significant, including impaired muscle contraction and reduced muscular endurance and performance [56,57]. According to current studies, microgravity-induced OS leads to bone loss and skeletal muscle atrophy in the musculoskeletal system (Table 1).

Microgravity-induced OS causes bone loss [58]. Lan et al. discovered that microgravity-induced OS caused an imbalance of bone metabolism, resulting in inhibited bone formation and enhanced bone resorption [59]. Microgravity-induced OS in the femur, evidenced by an increase in malondialdehyde (MDA) content and a decrease in total protein sulfhydryl content in the femur, leads to bone loss [60]. In vitro, microgravity also induced OS in osteoblasts, shown by a significant reduction in oxidized GSH and antioxidant enzymes, which reduced osteoblast differentiation and increased osteoclast formation [61]. Sun et al. reached the same conclusion [62]. Morabito et al. found that OS induced the impairment of the cytoskeleton structure, cell proliferation, and metabolism in osteoblasts [63]. Additionally, microgravity-induced OS caused the shortening of the primary cilia in osteoblasts. The injury on primary cilia led to increased expression and overactive channel of transient receptor potential vanilloid 4 (TRPV4), causing intracellular Ca^2+^ overload and OS, which eventually caused bone loss [64]. In summary, microgravity-induced OS disrupts bone metabolic homeostasis by decreasing osteoblast proliferation and differentiation, impairing cytoskeletal structure and metabolism, shortening the primary cilia of osteoblasts, and increasing osteoclast formation, which ultimately leads to bone loss.

Microgravity-induced OS causes skeletal muscle atrophy. The accumulation of monoubiquitinated lactate dehydrogenase (LDH) was found in muscles under simulated microgravity and spaceflight conditions in 2005. H_2_O_2_ induced the mono-ubiquitination of LDH-A in cells overexpressing LDH-A and ubiquitin, suggesting that simulated microgravity induced OS in skeletal muscles [70]. Biopsy samples of the vastus lateralis were collected after 48h unloading via unilateral lower limb suspension, and the OS response pathway was found to be the second-highest-ranked upregulated classical pathway [65]. A significant change was observed in flounder muscle in microgravity. The levels of antioxidant proteins (Gpx3, Gstm1, Gstm2, and Sod1) in flounder muscles were increased in spaceflight mice, suggesting that the disruption of antioxidant function contributed to OS in atrophied muscles [71]. Uchida et al. discovered that high levels ROS induced muscle atrophy via casitas B-lineage lymphoma-b (Cbl-b) expression activated by the ERK1/2 early growth response protein (Egr)-Cbl-b signaling pathway in microgravity [66]. Genchi et al., by sequencing muscle cells, found that H2A histone family member X (*H2AFX*), which is involved in many DNA damage metabolic pathways, was downregulated in microgravity [67,72]. The lack of H2AFX led to impaired redox homeostasis [73]. A number of studies on muscle atrophy due to microgravity-induced OS focused on the production of Nox2 and the reduction in Nrf2 levels. Mechanical unloading during microgravity led to skeletal muscle atrophy via increased ROS production by Nox2, as well as an impaired antioxidant system, affecting, e.g., sirtuin 1 (SIRT1) and SOD [68]. Nrf2 regulated antioxidant proteins, its expression was significantly reduced in microgravity, and the inhibition of Nox2 prevented the reduction in Nrf2 levels. The Nox2 inhibitor (gp91ds-tat) attenuated the unloading-induced increase in the p67phox subunit from Nox2, thereby attenuating the unloading-induced atrophy of halibut muscles [19]. In addition, computer prediction and RNA from the quadriceps muscles of spaceflight mice showed that the inhibition of integrin and Nrf2-mediated signaling in response to OS led to the loss of quadriceps muscles [69]. In vitro, microgravity downregulated the transcription of mitochondrial uncoupling protein 2 (*UCP2*), which is a sensor and antidote for ROS production by mitochondria in skeletal muscle cells [74]. Targeting the Nrf2/Nox2 pathway has emerged as one of the ideas for treating muscle atrophy caused by microgravity.

### 3.2. Effects of OS on the Cardiovascular System in Microgravity

Microgravity causes adaptive changes in the cardiovascular system. For instance, it turned the heart into a rounder shape [75]. Studies have shown that total cardiac work is lower during spaceflight [76]. Microgravity also significantly affects the structure and function of vessels. As an illustration, microgravity increased the vessel wall thickness and the intima–media area in the basilar artery and decreased the wall thickness of the femoral artery, which might be consequences of the modulation of the content of focal adhesions (FAs) in the vessels by microgravity [77]. In vitro, microgravity caused structural and functional changes in endothelial cells (ECs) and vascular smooth muscle cells (VSMCs) [78,79].

A majority of studies have shown that microgravity increases OS in the cardiovascular system, causing abnormalities of the myocardial and vascular structure and function (Table 2). Microgravity-induced OS had a negative impact on cardiovascular health [80,81]. In contrast, another study has shown that the cardiovascular system can adapt to microgravity without noticeable abnormalities [82]. Tahimic et al. found that cardiovascular oxidative damage was sex-dependent, and it was more pronounced in women [83].

Microgravity-induced OS causes myocardial abnormalities. Microgravity activated NADPH oxidase, increased ROS content, and decreased MDA expression in the heart [84]. This phenomenon was mediated by the inhibition of p47phox phosphorylation on Ser345 via the ERK1/2 and p38 pathways [85,86]. Ser345 is an important step in the initiation of NADPH oxidase activation [96]. By using engineered human heart tissues (EHTs), Mair et al. sequenced RNA from the tissues after spaceflight and found that they exhibited mitochondrial dysfunction and impaired lipid oxidation, with increased ROS production. The same study also revealed a potential link between OS and mitochondrial dysfunction in microgravity environments through computer simulations, which corresponded to the RNA sequencing results [87].

Microgravity causes increased superoxide in blood vessels, resulting in vascular abnormalities. In human umbilical vein endothelial cells (HUVECs), spaceflight was closely associated with upregulated thioredoxin-interacting protein (*TXNIP*) and downregulated OXPHOS genes, which triggered mitochondrial dysfunction. Both mitochondrial dysfunction and *TXNIP* overexpression contribute to a pro-oxidant environment, leading to OS and promoting DNA damage and inflammation [88]. Moreover, simulated microgravity activated NADPH enzymes and increased ROS in the cerebral vasculature [89,90,91]. ROS in the cerebral vasculature activated matrix metalloproteinase-9 (MMP-9), which triggered the upregulation of aquaporin-4 (AQP4), impairing the integrity of the blood–brain barrier (BBB) and creating vasogenic brain edema [92,93,97]. Increased superoxide levels in the basilar and carotid arteries in simulated microgravity resulted in an enhanced maximal contractile response [94,98]. The increase in superoxide levels was regulated by the ANGII/AT1 receptor signaling pathway [95]. Interestingly, it appears that not all blood vessels are influenced by microgravity. Some studies have found that simulated microgravity has no effect on superoxide levels in the mesenteric arteries and mesenteric smooth muscle cells [89,90,91]. In summary, microgravity-induced OS causes myocardial abnormalities via ERK1/2 and p38 pathways, enhances the maximal contractile response of vessels, and promotes DNA damage of the vein endothelial cells in the cardiovascular system. However, some studies have also shown that microgravity does not cause oxidative stress in some blood vessels. The effects of OS on the cardiovascular system in microgravity need to be explored further.

### 3.3. Effects of OS on the Brain in Microgravity

Microgravity modifies the structure and function of the brain. It was shown to move the body fluids toward the head, with microstructural changes in cerebral white matter and the redistribution of cerebral spinal fluid (CSF), developing a hydrocephalus-like structure, which damages brain tissue and affects brain function [8]. Once astronauts return to the ground, BBB permeability increases, which might lead to symptoms such as optic disc edema and cognitive dysfunction [99]. Spaceflight also decreased the expression of crucial genes involved in dopamine (DA) synthesis and degradation in the brain, as well as the D1 receptor [100]. This finding significantly contributes to explaining the dyskinesia and motor incoordination observed in astronauts resulting from exposure to spaceflight conditions. In addition, Marotta et al. created a brain-like organ composed of cortical and DA neurons, which serves as a model for studying neurons affected by multiple sclerosis and Parkinson’s disease. Brain-like organs were cultured on the International Space Station (ISS) for one month. Organoids grown in microgravity were found to have higher levels of gene expression associated with maturation and lower levels of gene expression associated with proliferation [101]. This means that in microgravity, cells develop faster and replicate less.

Several studies have shown that microgravity causes OS in the brain (Table 3). Microgravity-induced OS primarily alters cortical and hippocampal function, resulting in a neurodegenerative-like condition. The enrichment of mitochondrial metabolic proteins in the hippocampus of rats in simulated microgravity revealed that mitochondrial electron transport, oxidative regulation, fatty acid metabolism, ATP metabolism, and responses to OS were downregulated [102]. Some studies also found that microgravity increases the levels of Nrf2, SOD, and peroxides in the hippocampus, leading to neuroinflammation and learning and memory deficits [103,104,105]. 4-Hydroxy-2-nonenal (4-HNE) is one of various aldehydes produced during lipid peroxidation. It reacts with proteins, DNA, and phospholipids, acting as an inducer and mediator of OS [106]. Microgravity modulated the increase in 4-HNE in the cortex and hippocampus by increasing Nox2 protein levels, leading to OS and damage to brain tissue [18,20]. However, by performing measurements in spaceflight mice within 2 days of splashing down, Mao et al. found that pathways related to cell death, repair, inflammation, and metabolic stress were significantly modified, but there were no significant differences in 4-HNE levels [107]. The different levels of 4-HNE in these studies were probably related to the differences in the models. Moreover, Thy-1 protein in the hypothalamus was upregulated in microgravity. Thy-1 increased ROS and induced OS [108,109]. Microgravity causes OS in the brain, but the effects and mechanisms of microgravity-induced OS in the brain are still little well studied.

### 3.4. Effects of OS on the Immune System in Microgravity

Microgravity influences the proliferation, differentiation, activation, metabolism, and structure of immune cells, thereby impacting their normal functioning [110]. It was found that the total number of immune cells decreased but the number of neutrophils increased in microgravity, possibly because IL-8 induced the release of more neutrophils from the bone marrow [111]. Microgravity also affects the secretion of cytokines, including pro-inflammatory and anti-inflammatory cytokines. The release of pro-inflammatory cytokines such as TNF-α, IL-1, and IL-6 was increased in microgravity [112]. The microgravity environment impacts the differentiation and metabolism of immune cells. Spaceflight and simulated microgravity significantly reduced hematopoietic progenitor cell (HPC) differentiation, decreased macrophage numbers and M1/M2 polarization, and led to metabolic reprogramming [113]. Microgravity downregulated MHC class II molecules and CD56 on the surface of DCs, inhibiting the activity of T cells [114]. Moreover, microgravity altered the migration and killing function of immune cells. In microgravity, the killing ability of natural killer (NK) cells was decreased [115]. The phagocytosis function of macrophages was also modified [116].

Currently, there are fewer studies related to the effects of OS on immune cells in microgravity. Blood from astronauts showed mild but persistent OS due to the increased production of superoxide and nitric oxide in granulocytes [117]. Moreover, it exhibited glutathione peroxidase 1 (GPX1) downregulation. GPX1 protects cells from oxidative damage and modulates the immune response [118]. The detection of CD4^+^ T cells from three astronauts showed features of persistent DNA damage response, including OS, inflammation, and telomere aberrations [119]. Additionally, Kaufmann et al. exposed polymorphonuclear leukocytes to parabolic flight and found that adenosine limited microgravity-induced OS by upregulating adenosine A2A receptor function. This anti-inflammatory signal was stronger than the signal in the normal physiological situation and may limit further cytotoxic damage [120]. In contrast, parabolic flight experiments with human leukemia Jurkat cells and monoblastic U937 cells revealed that the latter had 100% adapted after 5 min of being exposed to microgravity. OS-related genes were detected in human Jurkat cells that showed almost no response to microgravity [121]. Further studies on OS in immune cells in microgravity are required.

### 3.5. Effects of OS on Other Organisms in Microgravity

Microgravity also induces OS in the liver, skin, lung, intestine, and reproductive system. Microgravity caused a rise in ferroportin in the liver [122]. Increased ferroportin protein levels contributed to OS in liver disease pathology [123]. Another study also showed the upregulation of a set of genes associated with OS in the livers of mice from the ISS, which led to liver damage after spaceflight [124]. Microgravity increased the susceptibility of ECs to OS after exposure to lipopolysaccharide, upregulated Nrf2 target genes in skin fibroblasts, and affected skin functions [125,126]. Liu et al. discovered that the genes involved in OS, DNA repair, and fatty acid oxidation were activated in WI-38 human embryonic lung cells in a spaceflight experiment [127]. Consistently, Wang et al. identified the upregulation of receptor for advanced glycation endproducts (RAGE) and the downregulation of peroxiredoxin 1 (PRDX1) in the lung tissues of rats in simulated microgravity. These were associated with OS and played important roles in microgravity-induced lung injury [128]. The ileal epithelial cells of mice in simulated microgravity exhibited an increased level of OS, which led to impaired ecological niche function of intestinal stem cells (ISCs) and caused dysbiosis [129]. Moreover, microgravity induced OS in Hodgkin’s lymphoma and neuroblastoma [130,131]. Interestingly, Berardini et al. observed a significant increase in mitochondrial O_2_^•−^ levels in TCam-2 spermatogonia in microgravity, and the expression of enzymes involved in redox homeostasis was also regulated to compensate for this. It was shown that these cells were able to trigger compensatory mechanisms that allowed them to overcome the regulation of microgravity [132]. In addition, microgravity had no significant effect on spongiosa [133]. Oxidative markers in saliva and serum were increased in a simulated microgravity environment, but there was no significant effect on periodontal parameters [134].

**Figure 1:**
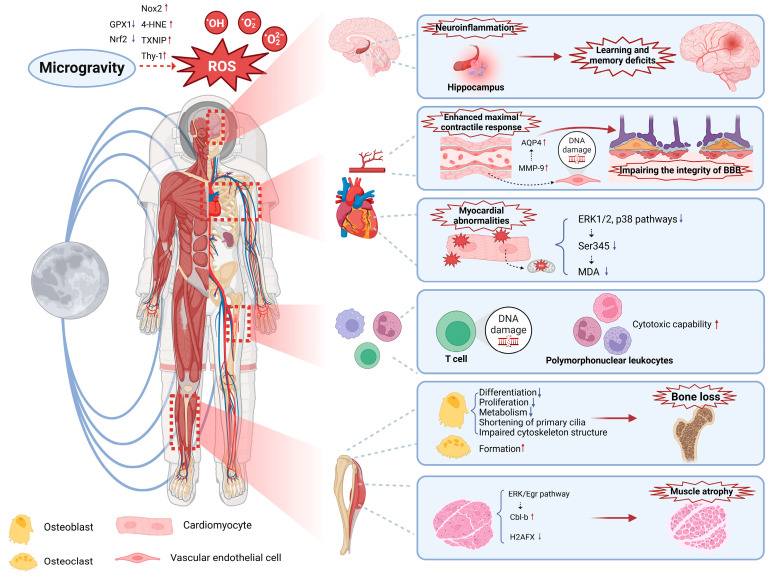
Effects of OS on the organism in microgravity. OS can cause dysfunction in the body, such as neuroinflammation, learning and memory deficits, myocardial and vascular abnormalities, impairment of the integrity of the BBB, oxidative damage to immune cells, bone loss, and skeletal muscle atrophy. BBB: blood–brain barrier.

In summary, OS is enhanced in microgravity and affects multiple systems, such as the musculoskeletal, cardiovascular, nervous, and immune systems, and so on, causing damage to the organism (Figure 1).

## 4. Therapy for OS on the Ground

The main therapeutic options for OS are reducing ROS, inhibiting downstream signaling, increasing antioxidant enzymes and their substrates, and repairing damage caused by OS. ROS produced due to outside environmental factors could be reduced by modifying lifestyle, such as avoiding smoking, radiation, and environmental pollution [15]. In organisms, ^•^OH is the key to oxidative damage. In vivo, the formation of ^•^OH, which is highly oxidizing, was prevented by reducing the production of O_2_^•−^ and H_2_O_2_ [25]. Fe^3+^Cyt c, acting as an antioxidant, also converts O_2_^•−^ into molecular oxygen [135]. Molecular hydrogen (H_2_) reduces the production of O_2_^•−^ by altering the direction of electron flow and neutralizing semiquinone radicals [136]. Moreover, environmental pollution led to the activation of NF-κB signaling in macrophages, which was directly inhibited by the addition of POD mimics. However, as H_2_O_2_ plays an important role in redox signaling and acts as a second messenger, its direct regulation led to abnormal signal transduction [137]. Phosphatidylcholine-specific phospholipase C (PC-PLC) was activated in this process. The inhibitor of PC-PLC, tricyclodecan-9-yl-xanthogenate (D609), is not an antioxidant but inhibited OS induced signal transduction [138]. The levels of antioxidant enzymes and their substrates were increased through exogenous supplementation or by activating antioxidant enzyme transcription factors. Dietary intake of vitamin C, vitamin E, and carotenoids enhanced the antioxidant capacity [139]. The expression of antioxidant enzymes like NQO1 and HO-1 was promoted through the activation of transcription factors, such as Nrf2, to neutralize oxygen free radicals and reduce oxidative damage [140,141]. The improvement in antioxidant enzyme activity, particularly that of quinone, increased the antioxidant capacity of cells [142]. Enzymes related to oxidative DNA damage repair, including FPG, AP endonucleases, and DNA polymerase, treated the damage caused by OS [34]. A considerable number of studies have been performed on treatments for OS on the ground, and the mechanism is clear.

## 5. Therapeutic Strategies for OS in Microgravity

**Figure 2:**
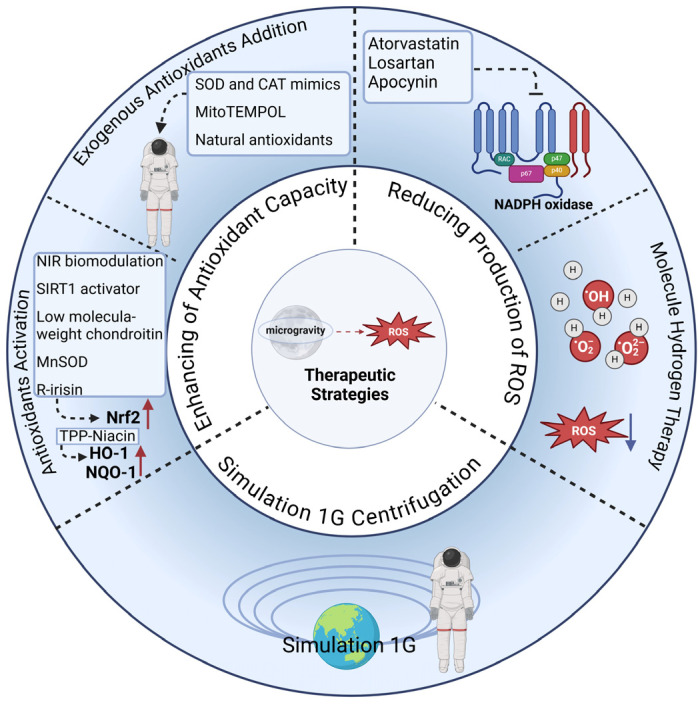
Therapeutic strategies for OS in microgravity. There are currently three strategies for treating microgravity-induced OS: the direct reduction of ROS by inhibiting oxidase activity or neutralizing peroxides, the enhancement of antioxidant capacity via exogenous antioxidant addition and inducing antioxidant activation induction, and exposure to 1G centrifugation. NIR: near-infrared.

There are three strategies for treating microgravity-induced OS: reducing the production of ROS, enhancing the antioxidant capacity, and simulating the terrestrial environment through 1G centrifugation. The production of ROS was directly reduced by inhibiting oxidase activity or neutralizing peroxides. Atorvastatin, losartan, and apocynin mitigated myocardial and vascular dysfunction by inhibiting NADPH oxidase, thereby reducing superoxide production [84,86,89,91]. Sun et al. used molecular hydrogen therapy to protect bone in simulated microgravity. In vivo, mice were fed hydrogen water (HW), and in vitro, cells were incubated with hydrogen-rich medium (HRM). It was found that hydrogen molecules reduced bone loss by suppressing osteoblast differentiation, osteoclast production, and ROS [62]. There are two ways to improve antioxidant capacity: exogenous antioxidant addition and antioxidant activation induction in vivo. A mimetic of SOD and CAT (EUK-134) was effective in attenuating skeletal muscle atrophy in microgravity [143]. Mito-TEMPO, a mitochondria-targeted antioxidant, reduced NADPH oxidase activity by attenuating mitochondrial fission via the upregulation of MFN1/2 and the downregulation of DRP1/FIS1, thereby attenuating enhanced vasoconstriction in cerebral arterioles of rats in simulated microgravity [91,95,144]. A number of natural antioxidants have also shown considerable potential for the treatment of microgravity-induced OS. Salidroside suppressed ROS production by activating the Nrf2/HO-1 signaling pathway and reduced microgravity-induced OS, thereby playing a bone-protective role and preventing bone loss [58]. Curcumin protected against bone loss by inhibiting ROS formation induced by microgravity and by enhancing osteoblast differentiation [60]. Natural flavonoids also protected the osteogenic potential of osteoblasts by reducing OS in microgravity [64]. Moreover, near-infrared (NIR) biomodulation, the SIRT1 activator, low-molecular-weight chondroitin, the mitochondrial antioxidant enzyme MnSOD, and r-irisin decreased microgravity-induced OS and reduced tissue damage via the activation of the Nrf2/Nox2 pathway [19,59,68,105,145]. TPP-Niacin also downregulated microgravity-induced ROS formation in ARPE19 cells (adult retinal pigment epithelial cells) by promoting the expression of antioxidant-related genes such as *HO-1* and *NQO-1* [146]. Currently, there is a treatment program for microgravity: the simulation of the terrestrial environment through 1G centrifugation. Kurosawa et al. exposed mice to 1G centrifugation in a space station and found that this partially attenuated liver injury due to OS in microgravity [124]. Mao et al. demonstrated that performing 1G centrifugation on the ISS effectively reduced endothelial cell damage and enhanced cellular organization and function compared with the microgravity group [107]. These studies provide new ideas for treating OS caused by microgravity (Table 4, Figure 2). However, there are few therapeutic strategies for microgravity-induced OS, and the mechanisms are unclear. More studies exploring the mechanisms and development of therapeutic options for microgravity-induced OS are needed.

## 6. Conclusions

This review documents current studies related to microgravity-induced OS as a significant factor in spaceflight causing dysfunction in the body as follows: bone loss and skeletal muscle atrophy, myocardial and vascular abnormalities, learning and memory deficits, oxidative damage to immune cells, liver damage, skin dysfunction, and lung damage. In the future, studying the mechanisms of microgravity-induced OS will not only contribute to our understanding of the effects on human health in space but also support the development of new therapeutic strategies. Some studies have also shown that cells rapidly adapt to microgravity without experiencing OS. The mechanisms of adaptation to microgravity remain to be further explored. Currently, the effects of microgravity-induced OS on organs are not yet well understood. To summarize, the mechanisms underlying microgravity-induced OS hold significant potential for further exploration, which would contribute to subsequent targeted therapy for microgravity-induced OS.

The exploration of therapeutic strategies offers potential solutions to reduce OS in microgravity. At present, there are three strategies for treating microgravity-induced OS: the direct reduction of ROS, the enhancement of antioxidant capacity, and exposure to 1G centrifugation. Overall, therapies for treating OS in microgravity remain underexplored, and further exploration will not only be essential for astronaut health during spaceflight but also provide new insights for the treatment of OS-related diseases on the ground.

In conclusion, this review presents a preliminary introduction and summary of the topic of microgravity-induced OS and therapeutic strategies pertaining to this condition. The study of microgravity-induced OS presents a promising avenue for future research. As space exploration progresses, research in this area will become vital to protecting astronaut health and advancing the field of space medicine. Subsequent research should focus on the mechanisms by which microgravity induces OS, the mechanisms by which microgravity-induced OS affects organs, and potential treatments for mitigating OS in space.

## Figures and Tables

**Table 1:** Overview of the effects of OS on the musculoskeletal system.

Type of Tissues/Cells	Model	Study Result	Refs.
Femur MC3T3-E1 cells	HLU RCCS	Cause bone loss by stimulating the Nrf2/HO-1 pathway	[58]
Femur	HLU	Alter bone microstructure and mechanical strength	[59]
Tibiae; Femur MC3T3-E1; RAW264.7 cells	HLU RWV	Reduce bone density and destroy bone structure in tibial, destroy mechanical strength in femur, reduce osteoblastic differentiation, and increase osteoclastogenesis	[60]
Human primary osteoblasts	RPM	Impair mitochondrial physiology as well as osteoblast function	[61]
Femur; lumbar vertebra MC3T3-E1; RAW264.7 cells	HLU RWV	Reduce bone mineral density, ultimate load, stiffness, and energy in femur and lumbar vertebra; reduce osteoblastic differentiation in MC3T3-E1 cells; induce osteoclastic differentiation and osteoclastogenesis in RAW264.7 cells	[62]
MC3T3-E1 cells	RPM	Alter cell cytoskeletal architecture, suppress cell proliferation rate and metabolism	[63]
Rat calvarial osteoblasts	RPM	Cause mitochondrial dysfunction and Ca^2+^ overload	[64]
Left vastus lateralis muscle	Unilateral lower limb suspension	Increase the mRNA expression of *HMOX*	[65]
L6 cells	ISS 3D clinostat	Cause muscle atrophy through Cbl-b expression activated by the ERK-Egr signaling pathway	[66]
H9c2 cells	ISS	Damage DNA via down regulation of *H2afx* expression	[67]
Gastrocnemius and soleus muscles	HLU	Cause skeletal muscle atrophy and impair mitochondrial energetics	[68]
Soleus muscles	HLU	Induced soleus muscle atrophy via elevation of Nox2	[19]
Quadriceps and soleus muscles	FLT	Decrease muscles wet weights	[69]

HLU: hindlimb unloading; ISS: International Staging System; RCCS: Rotating Cell Culture System; RPM: random positioning machine; RWV: the rotating wall vessels; FLT: flight.

**Table 2:** Overview of the effects of OS on the cardiovascular system.

Type of Tissues/Cells	Model	Study Result	Refs.
Ventricle	FLT	Contribute to cardiac dysfunction by altering the expression of genes regulating redox balance, cell cycle, and senescence	[81]
Hearts	HLU	Lead myocardial atrophy and dysfunction via increasing Rac1 activity	[84]
Heart tissues neonatal mouse cardiomyocytes	HLU RCCS	Promote myocardial abnormalities by facilitating p47phox phosphorylation via ERK1/2 and p38 pathways	[85]
Heart tissues	HLU	Induce myocardial dysfunction	[86]
EHTs	ISS	Induce mitochondrial dysfunction	[87]
HUVECs	FLT	Activate inflammatory responses, alter endothelial behavior, promote senesce	[88]
Cerebral and mesenteric VSMCs	HLU	Regulate cerebrovascular redox status and participate in vascular injury	[89]
Cerebral and mesenteric VSMCs	HLU	Result in cerebrovascular mitochondrial dysfunction	[90,91]
Brain HBMECs	HLU 3D clinostat	Induce BBB dysfunction via Rac1/Wave2/Arp3 signaling pathway	[92]
Brain	ISS	Indicate a disturbance of BBB integrity	[93]
Basilar and common carotid artery	HLU	Enhance maximal contractile response and impair endothelium-dependent relaxation	[94]
Basilar and common carotid artery	HLU	Enhance maximal contractile response and impair endothelium-dependent relaxation through an ANG II/AT1 receptor signaling pathway	[95]

VSMCs: vascular smooth muscle cells; EHTs: engineered human heart tissues; HUVECs: human umbilical vein endothelial cells.

**Table 3:** Overview of the effects of OS on the brain.

Type of Tissues/Cells	Model	Study Result	Refs.
Hippocampus	ISS	Increase in apoptosis	[93]
Hippocampus	HLU	Induce cognitive impairment by downregulation of the Sirt1/Nrf2 signaling pathway	[105]
Cortex; hippocampus	HLU	Cause learning and memory impairments	[104]
Hippocampus	HLU	Affect the function of hippocampus	[103]
Cortex	HLU	Induce endothelial damage and neurovascular remodeling	[18]
Cortex; hippocampus	HLU	Increase the likelihood of brain injury	[20]

**Table 4:** Overview of the therapeutic strategies for OS in microgravity.

Type of Tissues/Cells	Therapeutic Strategy	Treatment	Study Result	Refs.
Heart	Direct reduction of ROS	Atorvastatin	Inhibit Rac1 activation to attenuate myocardial atrophy	[84]
Heart		Losartan	Preserve cardiomyocyte size and prevent myocardial dysfunction by blocking Ser345 and NADPH oxidase activation	[86]
Cerebral VSMCs		Apocynin	Regulate cerebrovascular redox status with NADPH oxidase inhibition; promote recovery of mitochondrial function	[89,91]
Femur; lumbar vertebra MC3T3-E1; RAW264.7 cells		HW & HRM	Alleviate microgravity-induced bone loss	[62]
Cerebral VSMCs	Enhancement of antioxidant capacity	MitoTEMPOL	Promote recovery of mitochondrial function	[91]
Basilar and common carotid artery		Losartan	Through ANGII/AT1 receptor signaling pathway	[95]
Femur MC3T3-E1 cells		Salidroside	Mitigate bone loss induced by stimulating the Nrf2/HO-1 pathway	[58]
Tibiae; Femur MC3T3-E1; RAW264.7 cells		Ccurcumin	Preserve bone structure and mechanical strength by upregulating VDR expression in femurs	[60]
Rat calvarial osteoblasts		Natural flavonoid moslosooflavone	Mitigate loss of osteogenic potential of osteoblasts by protecting primary cilium	[64]
Hippocampus		PBM	Mitigate cognitive impairment through the activation of the Sirt1/Nrf2 signaling pathway, reduction in OS	[105]
Gastrocnemius and soleus		SRT2104	Preserve mitochondrial function to prevent skeletal muscle atrophy	[68]
Femur		LMWCSs	Protect against the bone loss related to reduce OS	[59]
Human osteoblasts		R-irisin	Prevent apoptotic death	[145]
ARPE19 cells		TPP-Niacin	Reduce ROS elevation by promoting the expression of antioxidant-related genes	[146]
Liver	Simulation of the terrestrial environment	1G centrifugation	Mitigate liver damage	[124]
ocular			Provide protection against changes	[107]

VDR: Vitamin D Receptor; PBM: Photobiomodulation; HW: Hydrogen Water; HRM: Hydrogen-Rich Medium; LMWCSs: Low-Molecular-Weight Chondroitin Sulfates.

## Abbreviations

The following abbreviations are used in this manuscript:

TME	Tumor microenvironment.
ROS	Reactive oxygen species.
OS	Oxidative stress.
SAS	Space adaptation syndrome.
SMS	Space motion sickness.
ICP	Intracranial pressure.
SANS	Spaceflight-associated neuro-ocular syndrome.
NADPH	Nicotinamide adenine dinucleotide phosphate.
NOXs	Nicotinamide adenine dinucleotide phosphate oxidases.
Nrf2	Nuclear transcription factor red lineage 2-related factor 2.
AREs	Antioxidant response elements.
HLU	Hindlimb unloading.
O_2_^•−^	Superoxide anion.
H_2_O_2_	Hydrogen peroxide.
^•^OH	Hydroxyl radicals.
8-oxoG	8-dihydroguanine.
OXPHOS	Oxidative phosphorylation.
SOD	Superoxide dismutase.
CAT	Catalase.
POD	Peroxidase.
APX	Ascorbate peroxidase.
vitamin C	Ascorbic acid.
vitamin E	Alpha-tocopherol.
GSH	Glutathione.
FPG	DNA glycosylase.
OGG1	8-oxoguanine DNA glycosylase.
DCs	Dendritic cells.
TRPV4	Transient receptor potential vanilloid 4.
LDH	Lactate dehydrogenase.
Cbl-b	Casitas B-lineage lymphoma-b.
D609	Tricyclodecan-9-yl-xanthogenate.
Egr	ERK1/2 early growth response protein.
H2AFX	H2A histone family member X.
SIRT1	Sirtuin 1.
UCP2	Uncoupling protein 2.
FAs	Focal adhesions.
ECs	Endothelial cells.
VSMCs	Vascular smooth muscle cells.
EHTs	Engineered human heart tissues.
HUVECs	Human umbilical vein endothelial cells.
TXNIP	Thioredoxin-interacting protein.
MMP-9	Matrix metalloproteinase-9.
AQP4	Aquaporin-4.
BBB	Blood–brain barrier.
CSF	Cerebral spinal fluid.
DA	Dopamine.
ISS	International Space Station.
MDA	Malondialdehyde.
4-HNE	4-Hydroxy-2-nonenal.
HPCs	Hematopoietic progenitor cells.
NK	Natural killer.
GPX1	Glutathione peroxidase 1.
RAGE	Receptor for advanced glycation endproducts.
PRDX1	Peroxiredoxin 1.
ISCs	Intestinal stem cells.
H_2_	Molecular hydrogen.
PC-PLC	Phosphatidylcholine-specific phospholipase C.
HW	Hydrogen water.
HRM	Hydrogen-rich medium.
NIR	Near-infrared.
